# Cucurbitacin E Induces Cell Cycle G2/M Phase Arrest and Apoptosis in Triple Negative Breast Cancer

**DOI:** 10.1371/journal.pone.0103760

**Published:** 2014-07-29

**Authors:** Yanjie Kong, Jianchao Chen, Zhongmei Zhou, Houjun Xia, Ming-Hua Qiu, Ceshi Chen

**Affiliations:** 1 Key Laboratory of Animal Models and Human Disease Mechanisms of Chinese Academy of Sciences and Yunnan Province, Kunming Institute of Zoology, Chinese Academy of Sciences, Kunming, Yunnan, China; 2 State Key Laboratory of Phytochemistry and Plant Resources in West China, Kunming Institute of Botany, Chinese Academy of Science, Kunming, Yunnan, China; 3 Kunming College of Life Science, University of Chinese Academy of Sciences, Kunming, Yunnan, China; University of Toronto, Canada

## Abstract

Triple negative breast cancer (TNBC) is a highly aggressive form of breast cancer resistant to many common treatments. In this study, we compared the effects of 12 phytochemical drugs on four cancer cell lines, and noticed that Cucurbitacin E (CuE) significantly inhibited TNBC cell growth by inducing cell cycle G2/M phase arrest and apoptosis. CuE reduced expression of Cyclin D1, Survivin, XIAP, Bcl2, and Mcl-1 in MDA-MB-468 and SW527, and within MDA-MB-468, CuE significantly increased activation of JNK and inhibited activation of AKT and ERK. Collectively, these results suggest that CuE may be a viable compound for developing novel TNBC therapeutics.

## Introduction

Breast cancer is a blanket term used to describe a variety of diseases, each with markedly different treatment options and prognosis for survival. While mortality rates from different forms of breast cancer have decreased on average over the last two decades, several variants remain resistant to common treatment options. In particular, ERα/PR/HER2 triple-negative breast cancer (TNBC), which accounts for 15–25% of breast tumors [1], often has poorest prognosis due to this particular cancer's insensitivity to traditional endocrine therapies and HER2 targeted therapies [2]. Poor prognosis of TNBC is exacerbated by a high rate of relapse in the 3–5 years following treatment, as well as the aggressive nature of the cancer. Though these factors have spurred a great deal of interest among oncologists, pathologists and medical researchers, few viable treatment options exist that have been shown to significantly improve survival rates for women with TNBC.

Given the state of research and prognosis of TNBC, there is a great need for novel therapeutics among the most promising are treatments derived from Cucurbitacins, which have been previously proposed to act as potential anti-tumor drugs. Cucurbitacins are a class of highly oxidized tetracyclic triterpenes present in several plants used in traditional Chinese medicine treatments (cucurbitaceous plants),which act by targeting the signal transducer and activator of transcription 3 (STAT3), fibrous-actin (F-actin), and cyclooxygenase-2 (COX-2) [3]. The first tested cucurbitacins, CuB and CuE, were previously found to promote anti-cancer activities in different types of cancers including breast cancer [3]. For example, administration of CuB and CuE in combination appeared to inhibit growth of ERα+ MCF-7 and ERα− MDA-MB-231 human breast cancer cell lines [4]. Later studies found that intraperitoneal administration of CuE significantly inhibited lung metastasis without affecting apoptosis or proliferation of 4T1 and MDA-MB-231 breast cancer cells [5], and also blocked breast tumor cell migration and invasion by modulating actin polymerization [5]. More recently, CuE was reported to suppress growth of Bcap37 and MDA-MB-231 breast cancer cells by inducing cell cycle arrest and apoptosis [6]. While each of these studies has yielded a variety of different findings—albeit promising ones—to date no study has thoroughly investigated the efficacy and functional mechanisms underlying CuE's different effects on TNBC.

Generally speaking, the existing evidence of CuE potential effects as a novel anti-cancer drug suggests that it functions by inducing cancer cell G2/M arrest [7]. Previous studies found that CuE (10 µM) increased the expression of cyclin-dependent kinase inhibitors p21 and p27 in MDA-MB-231 cells [6], and that CuE (0.5–1 µM) likewise up-regulated the expression of p53 and p21 proteins in the bladder cancer cell line T24 [7]. Similarly, administration CuE at 0.5–1 µM significantly inhibited the levels of pSTAT3 and CDK1 [7], while when administration at 50 nM to human leukemia HL-60 cells it increased the levels of peIF2 and p21 while also decreasing the level of CDK1 [8]. CuE also seems to induce apoptosis in several cancer cell lines [6]–[8], including the human oral squamous cell carcinoma cell line SAS (1.25–5 µM dosage) [9], and the human breast cancer cell lines Bcap37 and MDA-MB-231(1–10 µM) [6]. The exact mechanisms of these effects are not entirely clear and seem to vary considerably. For example, in the bladder cancer cell line T24, CuE administered at 0.5–1 µM induced apoptosis and triggered up-regulation of Fas/CD95, truncated BID (t-BID), apoptosis-inducing factor (AIF), and sequential activation of caspase-8, caspase-9, and caspase-3 [7]. CuE at 1–10 µM has also consistently decreased levels of the anti-apoptotic proteins XIAP, Survivin, and Mcl-1, and increased levels of the pro-apoptotic protein Bax in human leukemia HL-60 cells [8]. The diversity of these results suggest that both mitochondrial (intrinsic) and death receptor (extrinsic) apoptotic signaling pathways play roles in CuE-induced apoptosis.

To test the proposed functions of potential roles that CuE, as well as to investigate other potential roles of cucurbitacins, we extracted 12 different compounds for further testing: cucurbitacin E (CuE), kinoin B, and cucurbitacin L (CuL) from *Hemsleya delavayi var. yalungensis*(Cucurbitaceae), endecaphyllacin A, 23,24-dihydrocucurbitacin D, cucurbitacin B, 23,24-dihydrocucurbitacin B, 22-deoxocucurbitacin D, cucurbitacin I, and 22,23-dihydrocucurbitacin E from *H. endecaphylla* [10], 25-acetoxy-23,24-dihydrocucurbitacin F and B 23,24-dihydrocucurbitacin F from *H. jinfushanensis* [11]. After testing the potential activities of these compounds against several lines of cancerous cells, we found that administration of CuE resulted in marked anti-cancer activities in breast cancer lines, as well prostate and gastric cancer lines. Specifically, the IC_50_ of CuE was about 10–70 nM in five TNBC cell lines, and among the TNBC cell lines MAD-MB-468 and SW527, CuE significantly decreased cell viability, induced cell cycle G2/M phase arrest, and trigged apoptosis. CuE at concentration of 0.2 µM decreased the protein levels of CyclinD1, XIAP, Survivin, and Mcl-1.

## Materials and Methods

### Plant materials

Tubers of *H. delavayi* var. *yalungensis* were collected from Yalong, Sichuan Province, China, in 2006 (no specific permissions were required for these activities as the field studies did not involve endangered or protected species), and further study was then conducted at the Kunming Institute of Zoology and Kunming Institute of Botany (Kunming, Yunnan, China). A voucher specimen (No. KIB 2006-12-9) was previously deposited at the State Key Laboratory of Phytochemistry and Plant Resources at the Kunming Institute of Botany, Chinese Academy Sciences, before being identified by Prof. Wen-Jin Zhan, Penzhou Institute for Pharmaceutical Control, Sichuan.

### Extraction and isolation of compounds

To gather the necessary compounds for further testing, approximately 2.0 Kg of *H. delavayi var. yalungensis*air-dried and powdered tubers underwent methanol under reflux (5×10 L) extraction, after which the resulting solution was filtered. Once the combined filtrate was concentrated under vacuum, 317.7 g of residue was obtained, and then dissolved in 2L of water before being extracted with EtOAc (1 L×3) and n-butanol (1 L×3).The EtOAc extract (162.7 g) was subjected to silica gel column chromatography and eluted with a gradient system of CHCl_3_/MeOH (1∶0, 30∶1, 20∶1, 10∶1) that yielded fractions I−V monitored by TLC. Fraction II (2 g) was repeatedly chromatographed over silica gel using CHCl_3_/(Me)_2_CO (50∶1, 20∶1, 15∶1) as eluent, followed by a reversed-phase column (RP-18) developing with aqueous MeOH (60%→70%) to yield 167 mg of CuE, 54 mg of kinoin B, and 32 mg of cucurbitacin L, which were identified by comparing its spectroscopic profile with the previously published data.

### Cell lines and cell culture

All cell lines used in this study were purchased from the American Type Culture Collection (ATCC). MDA-MB-231, MDA-MB-468, SW527 were cultured in Dulbecco's Modified Eagle's Medium (DMEM). PC-3 was cultured in Ham's F-12 Medium. HCC1806, HCC1937 and NCI-N87 were cultured in RPMI-1640 medium. MCF7 was cultured in Minimum Essential Medium (MEM) with 0.01 mg/ml human recombinant insulin. All media were purchased from HyClone (Logan, UT) and supplemented with 10% FBS. All cells in the various media were maintained at 37°C with 5% CO_2_ in humidified atmosphere.

### Antibodies

The anti-PARP, Survivin, Mcl-1, XIAP, Bcl-2, Bcl-X_L_, p21, pSTAT3, STAT3, pAKT, AKT, pJNK, JNK, p-c-JUN, c-JUN, Cyclin D1 were obtained from Cell Signaling (Danvers, MA). The anti-Cyclin B1 antibody was from Abnova (Taipei, Taiwan). The anti-Cyclin E1 antibody was from Zymed (San Francisco, CA). The anti-p27 antibody was from Becton Dickinson (San Diego, CA). The anti-Caspase-3 and anti-cleaved Caspase-3 antibodies were from Imagenex (San Diego, CA). The anti-pERK, ERK, and GAPDH antibodies were from Santa Cruz (Santa Cruz, CA). The anti-β-actin antibody was obtained from Sigma (St. Louis, MO).

### Cell viability measurement

Cell proliferation was measured with a Sulforhodamine B assay (SRB, Sigma). In brief, MCF7, MDA-MB-468, PC-3, and NCI-N87 cells were seeded into 96-well plates at 2, 000 cells/well. After 24 h, the cells were treated with 12 cucurbitacins (10 µM) for 48 h, with DMSO serving as a negative control. The cells were then fixed with 100 µl 10% trichloro acetic acid for 60 m and then washed 5 times with deionized water. The cells were stained with 50 µl 0.4% (W/V) SRB in 1% acetic acid for 5 m, and then the plates were washed 5 times with 1% acetic acid and dried. Finally, 100 µl 10 mM Tris base was added to each well. Optical densities at 530 nm were measured at a spectrophotometric plate reader. The cell viability values at different drug dosages were plotted in EXCEL and IC_50_ values were obtained from the graphs.

### DNA synthesis

DNA synthesis of MDA-MB-468 and SW527 was measured with the Click-iT EdU microplate assay kit (Invitrogen, Carlsbad, CA) according to the manufacturer's protocols. Totally, we observed 10 fields randomly and counted the total number of cells and EdU positive cells respectively for each sample. The EdU positive cell number was divided by total cell number for each field. The resulting average percentage from the 10 fields was calculated and then plotted for further analysis.

### Cell cycle analysis

Adherent and detached MDA-MB-468 and SW527 cells were digested, harvested, and washed twice with PBS. Roughly 6×10^5^ cells were resuspended in 150 µl BD Cytofix/Cytoperm buffer solution (Cat#554722). After 20 m in 4°C, the cells were washed twice with BD Perm/Wash buffer (Cat. 554723) and incubated with 200 µl dying buffer (containing 0.1 mg/ml propidium iodide, 2 mg/ml RNaseA), incubated for 30 m at 37°C in the dark. The cells were then analyzed on an Accuri C6 flow cytometer (BD).

### Apoptosis

MDA-MB-468 and SW527 cells were treated with different concentrations of CuE for 24 h. Doxorubicin (Sigma) was used as the positive control. The cells were stained with anti-Annexin V antibody (eBioscience) and 7-AAD (Becton Dickinson) and then analyzed via flow cytometry.

### Western blotting (WB)

MDA-MB-468 and SW527 cells were treated with both different concentration of CuE or treated for different amounts of time, after which they were collected using a lysis buffer with a protease inhibitor cocktail (Roche Applied Science, Mannheim, Germany). The cell lysate was centrifuged and the resulting supernatant was collected, mixed with the sample buffer, and boiled for 5 m, after which the proteins were subjected to SDS-PAGE and transferred onto polyvinylidene fluoride (PVDF) membranes. The membranes were incubated with diluted primary antibodies, horseradish peroxidase (HRP) conjugated secondary antibodies (Jackson ImmunoResearch Laboratory, West Grove, PA), Western Lighting Chemiluminescence Reagent Plus (PerkinElmer Life Sciences, Shelton, CT) and then viewed on an ImageQuant LAS4000 Biomolecular imager (GE), in order to visualize the expression levels of specific proteins.

### Statistical analysis

All experiments were repeated at least three times to ensure accuracy. Final, the values are expressed as mean ± standard deviation (S.D.) and analyzed by student t test. The level of significance: * p<0.05, ** p<0.01, *** p<0.001.

## Results

### CuE is the most potent compound suppressing cancer cells

**Figure 1:**
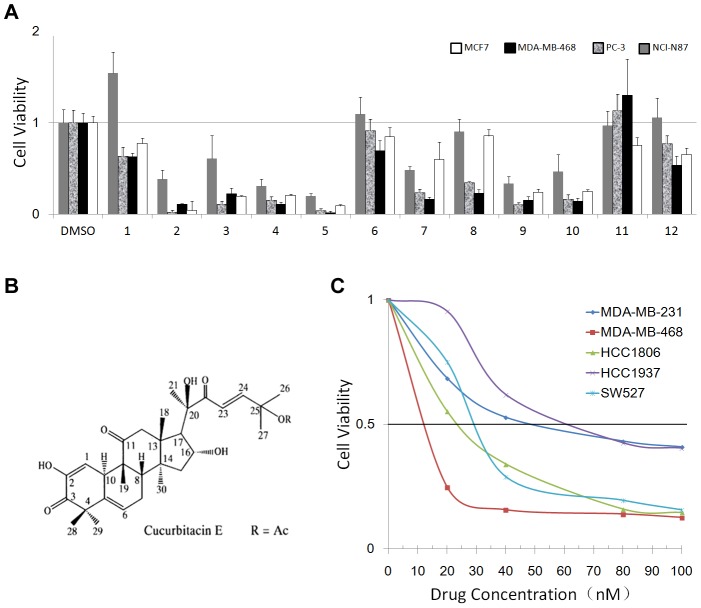
Identification of CuE as a potent anti-cancer compound in different cancer cell lines, including TNBC cell lines. A. Four different cancer cell lines were treated with 12 different cucurbitacins (10 µM) for 48 h. Cell viability was measured with the SRB assay. DMSO was used as the negative control. 1. Jinfushanencin B; 2. Cucurbitacin B; 3. 23, 24-dihydrocucurbitacin B; 4. 23,24-dihydrocucurbitacin D; 5. Cucurbitacin E; 6.22, 23-Dihydrocucurbitacin E; 7. 25-acetoxy-23,24-dihydrocucurbitacin F; 8. 23,24-dihydrocucurbitacin F; 9. Cucurbitacin L; 10. Kinoin B; 11. Endecaphyllacin A; 12. Elaeocarpucin C. Five potent anti-cancer compounds were labeled with underline. B. Chemical structure of CuE. C. Five different TNBC breast cancer cell lines treated with different concentrations (0–100 nM)) of CuE. Cell viability was measured with the SRB assay.

To identify anti-cancer compounds present in cucurbitacins, we treated four human cancer cell lines (ERα positive breast cancer cell line MCF7, TNBC cell line MDA-MB-468, prostate cancer cell line PC-3, and gastric cancer cell line NCI-N87) with 12 individual cucurbitacins at concentration of 10 µM for two days, and then measured the resulting cell viability via SRB assays. Of the 12 tested compounds, CuE and four other compounds (CuB, CuL, 23, 24-dihydro CuD, and 24-acetoxy-23, 24-dihydro CuF) resulted in dramatic reduction in cell viability in each of the four cancer cell lines when compared to the control DMSO (Figure 1A). We selected these five compounds with different dosages and treated them in five TNBC cell lines (MDA-MB-468, MDA-MB-231, HCC1806, HCC1937, and SW527) by the SRB assay. We found that CuE's half maximal inhibitory concentrations (IC_50_) was the lowest of the five tested compounds. (Table 1)

**Table 1:** IC_50_ of five cucurbitane compounds in six cancer cell lines.

Cell lines	CuB	CuE	CuL	23,24-dihydro-cucurbitacin D	25-acetoxy-23,24-dihydro-cucurbitacin F
MCF7	0.12	0.05	0.27	0.18	0.98
MDA-MB-468	0.12	0.02	0.05	0.08	0.26
PC-3	0.02	0.02	0.06	0.08	0.44
NCI-N87	0.31	0.05	0.11	0.25	0.76
MG63	0.04	0.04	0.05	0.08	0.2
MDA-MB-231	0.18	0.03	0.08	0.13	0.21

CuE (Fig. 1B) exhibited the most potent anti-cancer effects in terms of IC_50_ in the cancer cell lines. The compound appeared to have similar effects in osteosarcoma MG63 and TNBC MDA-MB-231 cell lines (Table 1). Given the strong effects of CuE, we further tested it against five TNBC cell lines (MDA-MB-468, MDA-MB-231, HCC1806, HCC1937, and SW527) and found that CuE inhibited the growth of all five TNBC lines in a dose-dependent manner (Fig. 1 C), with the IC_50_ being about 10–70 nM. Overall, the MDA-MB-468 line appeared to be the most sensitive TNBC cell line in regards to the effects of CuE (Fig. 1C).

### CuE inhibits DNA synthesis in MDA-MB-468 and SW527 TNBC cell lines

**Figure 2:**
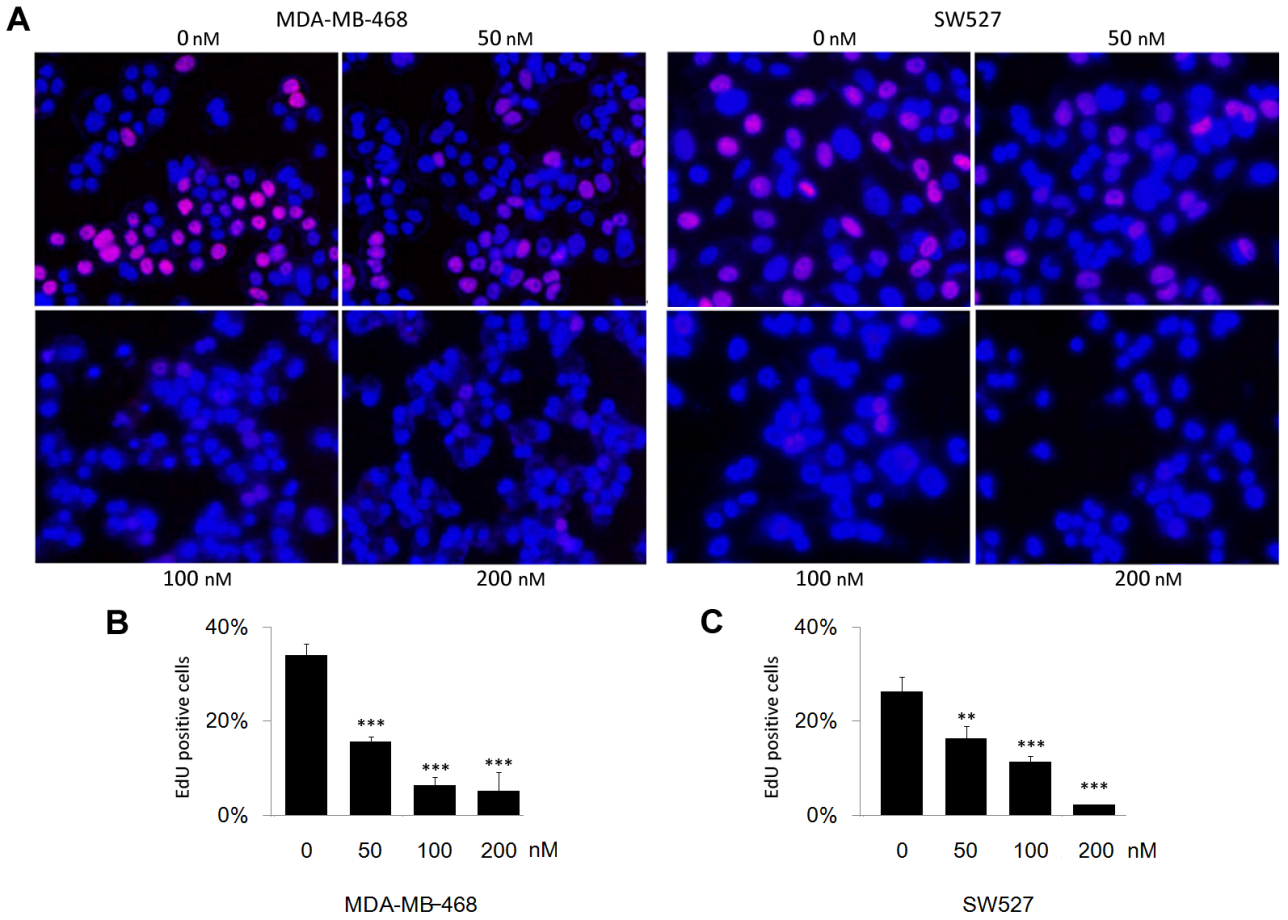
CuE suppresses DNA synthesis in MDA-MB-468 and SW527 TNBC cell lines. A. CuE (0–50–100–200 nM) was used to treat MDA-MB-468 and SW527 cells for 24 h. DNA synthesis was measured by the EdU assay. B. Quantitative data of MDA-MB-468. Percentage of EdU positive proliferating cells vs. total cells is shown. *** p<0.001, student t-test. C. Quantitative data of SW527. Percentage of EdU positive proliferating cells vs. total cells is shown. ** p<0.01, *** p<0.001, student t-test.

To investigate whether CuE inhibits cell proliferation in TNBC cell lines, we used an EdU assay to measure the DNA synthesis [12]. Results showed that CuE dramatically inhibited DNA synthesis in both MDA-MB-468 and SW527 cell lines in a dosage dependent manner (Fig. 2).

### CuE causes the cell cycle G2/M arrest in MDA-MB-468 and SW527 TNBC cell lines

**Figure 3:**
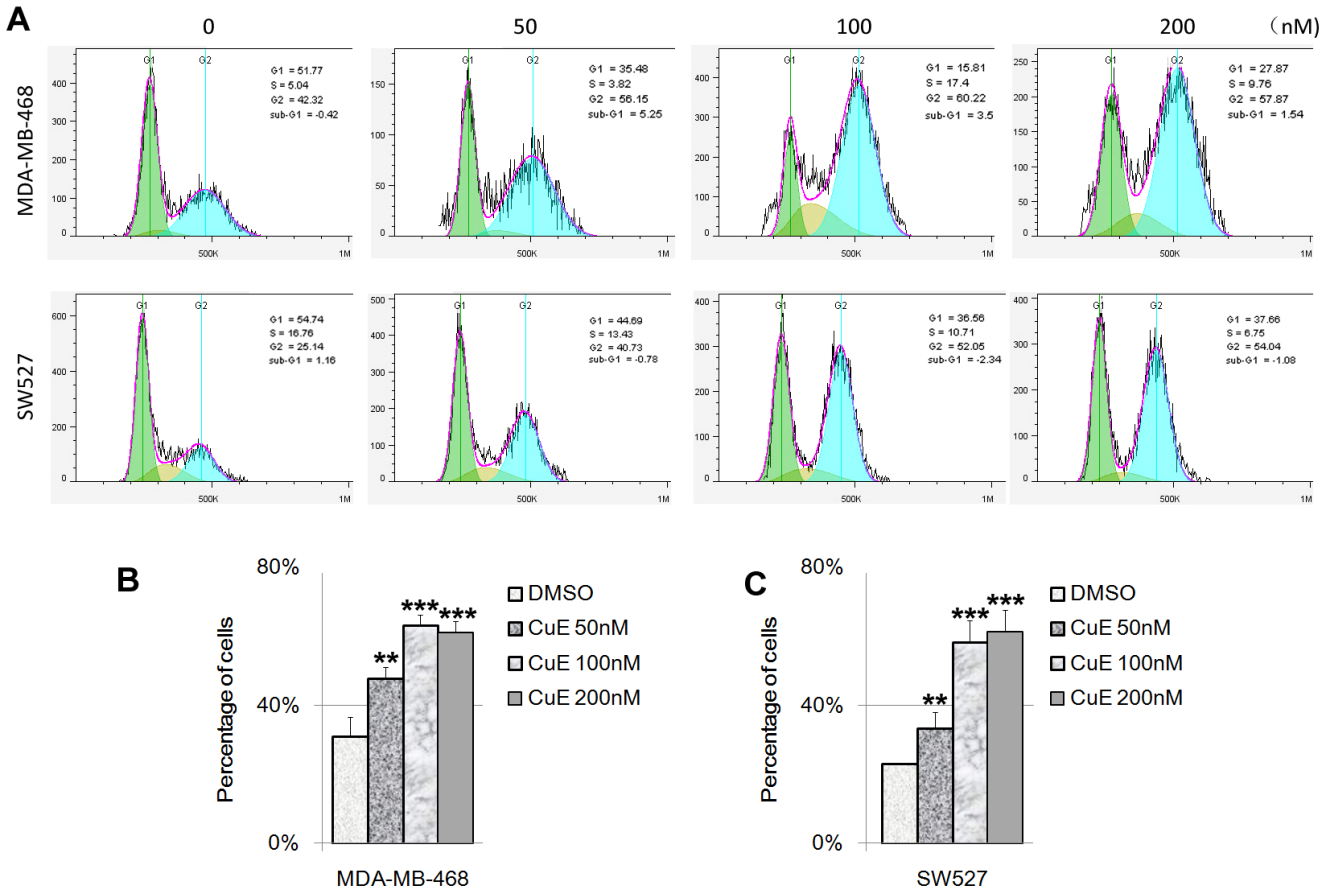
CuE induces cell cycle G2/M arrest in MDA-MB-468 and SW527 TNBC cell lines. A. MDA-MB-468 and SW527 cells were treated with CuE (0–50–100–200 nM) for 24 h. Cells were stained with PI and analyzed by flow cytometry. The cell cycle graph was analyzed by FlowJo software (version 7.6). B. CuE significantly increased the percentage of G2/M phase MDA-MB-468 cells compared to DMSO. ** p<0.01, *** p<0.001, student t-test. C. CuE significantly increased the percentage of G2/M phase SW527 cells compared to DMSO. ** p<0.01, *** p<0.001, student t-test.

To test whether CuE modulates the cell cycle among TNBC cells, we treated MDA-MB-468 and SW527 cells with CuE at several different dosages (0, 50, 100, and 200 nM) for 24 h, after which we measured cell cycle distribution. Results showed that administration of CuE significantly increased the percentage of cells in the G2/M phase in a dosage dependent manner in both MDA-MB-468 and SW527 cell lines (Fig. 3), with the G2/M arrest induced by CuE reached its peak at 100 nM. Further increases in dosages of CuE (200 nM) did not appear to significantly increase the percentage of cells in the G2/M phase in both MDA-MB-468 and SW527 lines.

### CuE induces apoptosis in MDA-MB-468 and SW527 TNBC cell lines

**Figure 4:**
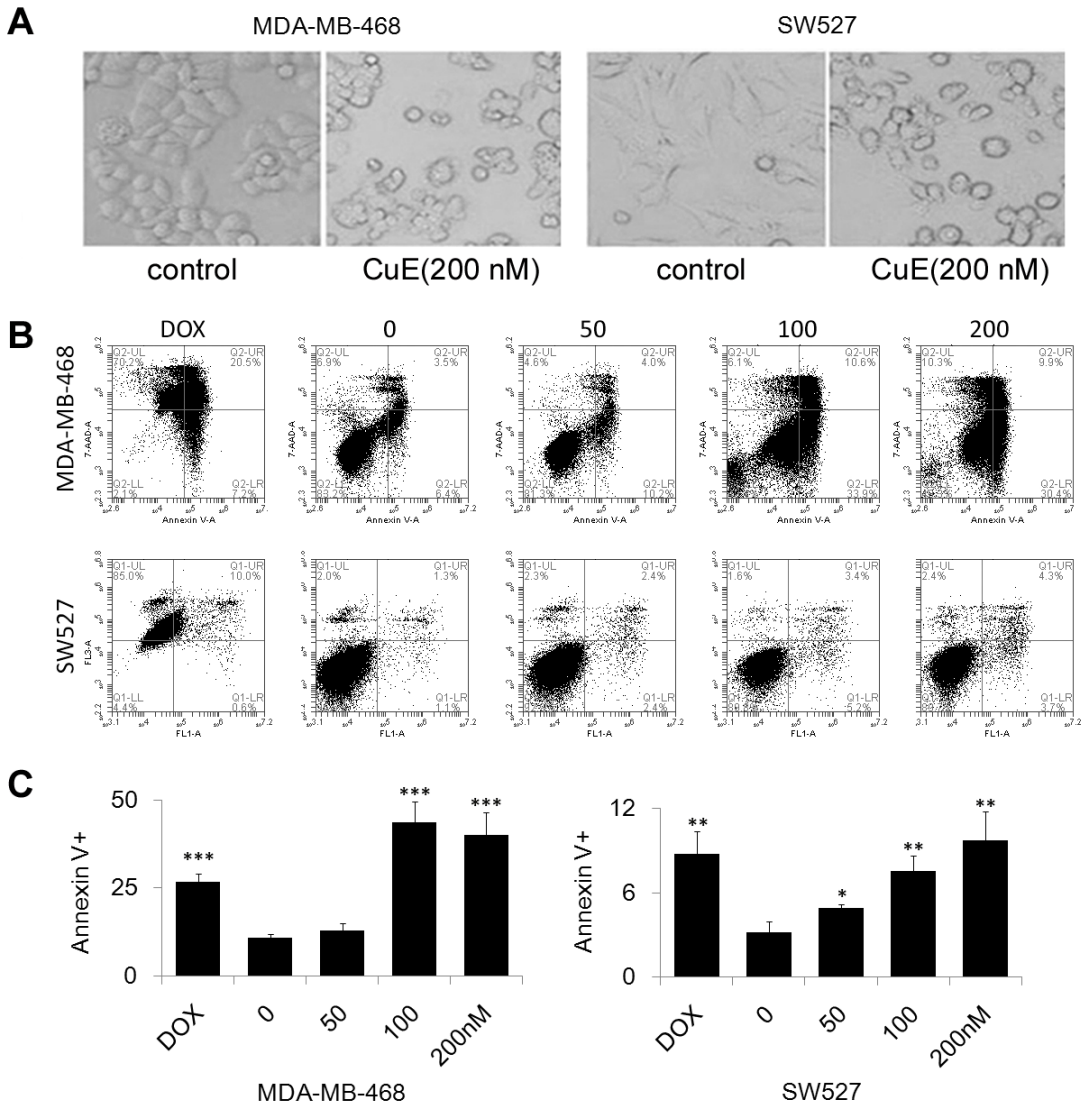
CuE induces apoptosis in MDA-MB-468 and SW527 TNBC cell lines. A. Cell morphology changed dramatically when MDA-MB-468 and SW527 cells were treated with CuE (200 nM) for 24 h. B. MDA-MB-468 and SW527 cells were treated with CuE (0–50–100–200 nM) for 24 h. Cells were stained with Annexin V/7AAD and analyzed by flow cytometry. Doxorubicin was used as a positive control. C. Quantitative data of panel A. Percentage of Annexin V-positive cells is shown. * p<0.05, ** p<0.01, *** p<0.001, student t-test.

**Figure 5:**
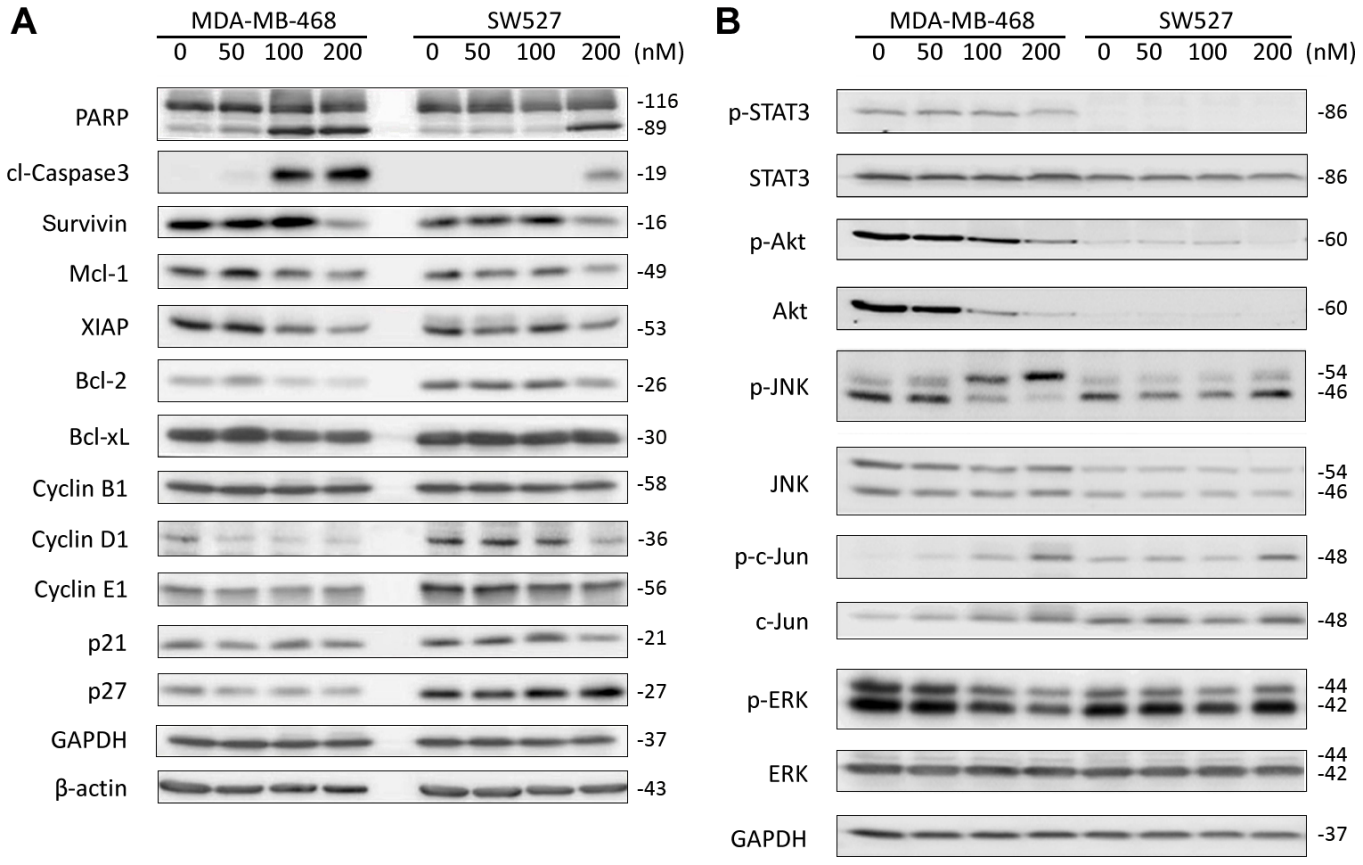
CuE modulates the expression levels of multiple cell cycle, apoptosis, and signaling regulators in MDA-MB-468 and SW527 TNBC cell lines. A. MDA-MB-468 and SW527 cells were treated with CuE (0–50–100–200 nM) for 24 h. Cell lysate were collected for WB, to detect PARP, cleaved Caspase-3, Survivin, Mcl-1, XIAP, Bcl-2, Bcl-xL, Cyclin B1, Cyclin D1, Cyclin E1, p21, and p27. β-actin and GAPDH were used as loading controls. B. MDA-MB-468 and SW527 cells were treated with CuE (0–50–100–200 nM) for 24 h. pSTAT3, total STAT3, pAKT, total AKT, pJNK, total JNK, p-c-Jun, total c-Jun, pERK and total ERK were examined by WB. GAPDH was used as a loading control.

After 24 h treatment with 200 nM of CuE, MDA-MB-468 and SW527 cells became round, detached, and formed apoptotic bodies (Fig. 4A), indicating that the cancerous cells underwent apoptosis following CuE treatment. We further measured apoptosis of MDA-MB-468 and SW527 cells via Annexin V/7-AAD staining and flow cytometry with doxorubicin serving as a positive control. Administration of CuE (100–200 nM) was found to significantly increase the Annexin V positive apoptotic cells in both MDA-MB-468 and SW527 lines (Fig. 4B–C),and also induce the cleavage of Caspase-3 and PARP in both cell lines (Fig. 5A).

### CuE modulates the expression levels of multiple cell cycle, apoptosis, and signaling regulators in MDA-MB-468 and SW527 TNBC cell lines

After noting that CuE induced cell cycle G2/M arrest and apoptosis, we further examined the protein levels of both the cell cycle and apoptosis regulators using WB. In both MDA-MB-468 and SW527 cells, CuE (100–200 nM) treatment for 24 h dramatically decreased the protein levels of Cyclin D1, but not Cyclin B1 or Cyclin E1 (Fig. 5A). Likewise, within both lines, CuE (100–200 nM) did not increase the protein levels of p21 and p27. Concurrently, CuE administration dramatically decreased the levels of several anti-apoptotic proteins, including Survivin, Mcl-1, XIAP, and Bcl-2, though not Bcl-X_L_.

Given the effects of CuE on MDA-MB-468 and SW527 cells, we further investigate whether CuE actually modulates the activities of STAT3, AKT, ERK, and JNK in these two cell lines. MDA-MB-468 was the most sensitive TNBC cell line to CuE administration with dosages of 100–200 nM decreasing levels of pSTAT3, pERK, pAKT and total AKT (Fig. 5B). Moreover, in this cell line, the administration of CuE also increased levels of pJNK and p-c-Jun (Fig. 5B). However, in SW527 cells, we did not detect the pSTAT3, pAKT and total AKT proteins, and CuE did not appear to dramatically change the levels of pERK, pJNK and p-c-Jun (Fig. 5B).

## Discussion

Despite numerous advances in cancer treatments, the resistance of certain forms breast cancer, especially TNBC, the lack of effective treatments has prompted a great deal of research into novel anti-cancer drugs derived from natural compounds. The cucurbitacin family has been an attractive target in this search, as they have been previously reported to exhibit strong anti-cancer effects in several different types of cancers [13], [14]. Within cucurbitacins, both CuB and CuE were found to exert a cytotoxicity effect in a number of breast cancer cell lines, including MDA-MB-231, ZR-75-1, BT474, MCF7, and SKBR3 [13]–[16]. Though these results were promising, little research has been done to delineate the underlying effects of CuE on TNBC. In this study, we tested 12 cucurbitacins from three *Hemsleya* species and compared their cytotoxic effects on four different types of cancer cell lines. Our results showed that CuE exerts the most potent anti-cancer effects, with the administration of CuE significantly decreased cell viability in five TNBC cell lines at low concentrations (IC_50_<100 nM). Furthermore, CuE induced cell cycle G2/M arrest and apoptosis in MDA-MB-468 and SW527 TNBC cell lines. Finally, CuE modulated cell cycle protein Cyclin D1, anti-apoptotic proteins Survivin, Mcl-1, XIAP, and Bcl-2, as well as several signaling pathways such as pSTAT3, pERK, pJNK, and pAKT in the most sensitive TNBC cell line.

Our findings strongly suggest that CuE may be a promising candidate developing novel TNBC therapeutics, though we also noted that four other cucurbitacin compounds (CuB, CuL, 23, 24-dihydro CuD, and 24-acetoxy-23, 24-dihydro CuF) also exhibited strong cytotoxic effects on six cancer cell lines (Table 1). Previous studies reported that CuB could induce apoptosis in pancreatic cancer cells [17], hepatocellular carcinoma cells [18], melanoma cells [19], breast cancer cells [20], colon cancer cells [21], and laryngeal squamous cell carcinoma [22]. However, the anti-tumor activities of our other tested compounds CuL, 23, 24-dihydro CuD, and 24-acetoxy-23, 24-dihydro CuF have not been thoroughly examined. Our results suggest that these natural products induce anti-tumor activity in different types of cancers, potentially indicating they may be useful targets for further research into novel therapeutics even though their anti-cancer effects were not as dramatic as those of CuE.

A key hurdle in developing novel cancer treatments is elucidating the underlying molecular mechanisms for compounds that exhibit anti-cancer effects. The current study greatly extends our understanding of the molecular mechanism by which CuE inhibits TNBC, wherein CuE induced cell cycle G2/M arrest in MDA-MB-468 and SW527 cells. Earlier reports showed that CuE caused T24 bladder cancer cell G2/M arrest through STAT3/p53/p21 signaling pathway [7], but the functional concentration for CuE affecting cells in the T24 line was as dosages of 0.5–1 µM. Similarly, administration of 10 µM of CuE caused an increased expression of p21 and p27 in MDA-MB-231 cells. Curiously, we observed no such up-regulation of p21 and p27 in MDA-MB-468 and SW527 cells when using concentrations of 200 nM of CuE. Though there may confounding factors, it seems that the expression change of p21 and p27 by CuE may in part be due to a dosage or cell line dependent effect. Indeed, we observed a down-regulation of Cyclin D1 by CuE in both MDA-MB-468 and SW527 cells. However, Cyclin B1 plays more important role in G2/M phases than Cyclin D1 does. In our study, CuE did not significantly down-regulate the expression of Cyclin B1 in MDA-MB-468 and SW527 cells, suggesting that CuE may cause G2/M arrest through other proteins in addition to Cyclin D1.

In addition to the noted effect of G2/M cell cycle arrest, CuE (100–200 nM) also induced apoptosis in MDA-MB-468 and SW527 cells. In previous studies, CuE (1–10 µM) inhibited the pSTAT3 and induced apoptosis in human breast cancer cell lines Bcap37 and MDA-MB-231 [6] and decreased the levels of the anti-apoptotic proteins XIAP, Survivin, and Mcl-1, and increased the level of Bax in human leukemia HL-60 cells [8]. Moreover, higher dosages of CuE (0.5–1 µM) induced the up-regulation of Fas/CD95, truncated BID (t-BID), AIF, and sequential activation of caspase-8, caspase-9, and caspase-3 in T24 bladder cancer cells [7]. In this study, we demonstrated that CuE at somewhat lower concentrations (100–200 nM) decreased the expression levels of Survivin, XIAP, Bcl2, and Mcl-1 in MDA-MB-468 and SW527 cells.

Considering both our current results and those from previous studies, it is plausible to assume that CuE modulates the expression of cell cycle and apoptosis regulators by interfering with key cancer related signaling pathways, such as Jak-STAT, PI3K-AKT, and Raf-MAPK. We demonstrated that CuE (200 nM) inhibited the pSTAT3 in MDA-MB-468 cells (Fig. 5B). Similar studies found that CuE (100 nM) consistently inhibited pSTAT3 in the PANC-1 pancreatic cancer cell line [17] and the ES-2 ovarian cancer cell line [23]. Additionally, CuE (10 nM) blocked VEGFR2-mediated Jak2-STAT3 and pERK signaling pathways in HUVEC cells [24]. For the first time, we noticed that CuE (100–200 nM) dramatically decreased the levels of pERK, pAKT, and total AKT in MDA-MB-468 cells. Similarly, CuB (0.1–1 µM) inhibited 12-O-tetradecanoylphorbol 13-acetate (TPA) induced pERK and pAKT in HepG2 cells [25]. We also found that CuE (100–200 nM) dramatically increased the levels of pJNK and p-c-Jun in MDA-MB-468 cells (Fig. 5B), though CuE has never previously been reported to activate the JNK-c-Jun pathway. CuB (100 nM) likewise activated pJNK and p-c-Jun in U87 and T98G glioblastoma cell lines [26] and CuI (200 nM) activated pJNK and p-c-Jun in B leukemic cells [27]. Taken together, these results suggest that the STAT3, ERK, AKT, and JNK/c-Jun signaling pathways may be targets for CuE in a subtype of TNBC.

In conclusion, our study on the 12 cucurbitacins found that CuE was the most potent cytotoxic compound among five active compounds that were shown to exert anti-cancer effects on several different cancer cell lines. CuE decreased cell viability in multiple TNBC cell lines and also induced G2/M cell cycle arrest and apoptosis in MDA-MB-468 and SW527 TNBC cell lines. The mechanism by which CuE inhibits TNBC may potentially be caused by down-regulation of Cyclin D1, Survivin, XIAP, Bcl2, and Mcl-1, the inactivation of STAT3, AKT and ERK, and the activation of JNK. Given the possibilities underpinning these different results, there are many potential mechanisms or effects of CuE and the other active compounds we tested that must be examined further. Such investigations may yield new avenues in the development of novel cancer treatments. However, our findings strongly support CuE as one of the most promising target for further investigation and development of novel therapeutics, especially towards TNBC.
